# Genotypic diversity of multi- and extensively drug-resistant *Mycobacterium tuberculosis* in Iran: a systematic review and meta-analysis

**DOI:** 10.1186/s12879-025-12299-y

**Published:** 2025-12-15

**Authors:** Mansour Kargarpour Kamakoli, Ehsan Zare BanadKoki, Mehran Nakhaeizadeh, Ava Behrouzi, Nasim Ebrahimifard, Shima Hadifar

**Affiliations:** 1 Department of Mycobacteriology and Pulmonary Research, Pasteur Institute of Iran, Tehran, Iran; 2 Department of Microbiology, Faculty of Medicine, Shahed University, Tehran, Iran; 3 Modeling in Health Research Center, Institute for Futures Studies in Health, Kerman University of Medical Sciences, Kerman, Iran; 4 Department of Microbiology, TeMS.C., Islamic Azad University, Tehran, Iran; 5 Department of Bacteriology, Pasteur Institute of Iran, Tehran, Iran

**Keywords:** *Mycobacterium tuberculosis*, Meta-analysis, Multidrug-resistant, Extensively drug-resistant, Genotype, Iran

## Abstract

**Background:**

Monitoring the genetic diversity of the drug-resistant *Mycobacterium tuberculosis* (Mtb) population contributes to gaining better insight into the bacterial dynamics and informing effective strategies to tackle the challenge of drug resistance. This meta-analysis study investigates the pooled prevalence of Mtb genotypes among Iranian multidrug-resistant (MDR)/extensively drug-resistant (XDR)-TB patients.

**Methods:**

We performed a systematic search across repositories, including PubMed, Scopus, Web of Science, and Iranian databases, to find studies reporting Mtb genotype prevalence among Iranian MDR/XDR-TB patients through 31 August 2024. Random-effects or fixed-effect models were used to evaluate the pooled proportion. Statistical analysis was conducted using a meta package in R software.

**Results:**

In this meta-analysis, 26 out of 34 included studies focused on MDR genotypes, while the remaining studies addressed the prevalence of pre-/XDR genotypes. The prevalence results for MDR genotypes showed that Haarlem (28.5%, 95% CI: 24.6–32.8), Beijing (25.6%, 95% CI: 22.5–28.9), and URAL (23.8%, 95% CI: 13.3–38.9) were the most predominant genotypes. Genotypes with the lowest proportion included MANU, T, and Uganda ( < 8%). The highest prevalence of pre-/XDR genotypes was identified for Beijing (57.6%, 95% CI: 35.8–76.8) and Haarlem (30.2%, 95% CI: 9.8–63.3), respectively.

**Conclusions:**

Based on our findings, Haarlem and Beijing were highlighted as the dominant circulating genotypes among Iranian MDR- and pre-/XDR-TB cases, respectively. As the relationship between these genotypes and high levels of treatment failure has been documented, local tracking dynamics of drug-resistant Mtb population structure are critical to improving our ability to manage local MDR/XDR-TB.

**Supplementary information:**

The online version contains supplementary material available at 10.1186/s12879-025-12299-y.

## Introduction

The management and control of tuberculosis (TB), caused by *Mycobacterium tuberculosis* (Mtb), continue to pose a global health challenge. Despite the global effort, the rate of decrease in TB incidence has been 1.6–2% per year [1]. The ongoing increase in the number of individuals diagnosed with multidrug-resistant (MDR), defined as resistance to at least isoniazid and rifampicin, pre-extensively drug-resistant –TB (pre-XDR), defined as MDR-TB with resistance to a fluoroquinolone, and extensively drug-resistant (XDR)-TB, which is MDR-TB having also resistance to a fluoroquinolone, bedaquiline, and/or linezolid [2], exacerbated the problem, particularly in resource-limited settings [3]. In 2021, the rate of increase in drug-resistant TB (DR-TB) was 6.4%. XDR-TB cases are on the rise, with 13,068 new cases reported across 81 countries. By 2022, the global proportion of MDR/XDR-TB was estimated to be 18%. [1, 4]. Notably, the incidence and mortality rate of DR-TB presented significant geographical heterogeneity [3].

In Iran, situated at the crossroads of high TB burden regions, the pooled prevalence of MDR-TB from 1981 to 2019 was reported at 12.31% and remains a critical concern [5]. This situation is further complicated by the diverse genetic landscape of *Mtb* strains circulating within the country, which can influence pathobiological properties, control measures, and specific clonal expansion within geographical regions [6–10]. Furthermore, certain populations have documented a relationship between genotype and treatment outcome [11, 12].

In the Iranian population, Beijing and Haarlem genotypes have been identified with notable prevalence among MDR-TB cases [13]. However, the recent increase in the circulation of NEW-1 and CAS genotypes, both known for their potential to acquire drug resistance [14, 15], underscores the necessity of local tracking of DR-Mtb population dynamics to identify specific pathobiological characteristics and also improve the ability to manage local MDR/XDR-TB.

Despite the growing body of research on Mtb epidemiology in Iran, a comprehensive and up-to-date analysis of DR strains diversity, particularly focusing on the genotypes of MDR and XDR Mtb, is lacking.

This systematic review and meta-analysis advances prior reviews, many of which focused on overall MDR-TB prevalence or single genotypes, by synthesizing evidence across studies to provide a consolidated overview of genotypic diversity among MDR- and pre-/XDR-TB cases in Iran.

## Methods

The protocol for this systematic review and meta-analysis was registered on the PROSPERO database with the registration number CRD42020223433. The search protocol adhered to the Preferred Reporting Items for Systematic Reviews and Meta-analyses (PRISMA) guideline [16] in the current study.

## Literature search strategy

A comprehensive literature search was conducted across international and regional databases—including PubMed, Scopus, Web of Science, and Iranian scientific repositories—covering publications up to 31 August 2024. The following terms with the connectors “AND” and “OR” were used for the study searches: “*Mycobacterium tuberculosis*,” “tuberculosis,” “multidrug resistance,” “MDR,” “XDR,” “drug-resistant,” “molecular typing,” “genetic diversity,” “genotyping, and “Iran”. Persian-language articles were retrieved using the same search strategy with Persian keywords in the relevant Iranian databases. The titles and abstracts of papers selected by our primary search were further examined for full-text review. Moreover, the literature searches were supplemented by checking the reference lists of the collected papers to identify citations not captured in the utilized database. Two authors independently performed the literature searches.

## Study selection

Eligible publications were selected based on the following criteria:

1) Presented the prevalence of MDR- and/or pre-/XDR-Mtb genotypes; 2) determined the genotypes based on Spoligotyping, mycobacterial interspersed repetitive unit-variable number tandem repeat (MIRU-VNTR) typing, IS6110-RFLP, and Whole Genome Sequencing (WGS) methods; 3) provided clear data on genotype frequencies; and 4) reported data from Iran.

We excluded studies if they were restricted to reporting the prevalence of drug-susceptible Mtb genotypes. Studies provided incomplete data or failed to present it clearly. Additionally, we excluded studies that were published as case reports, meta-analyses, and systematic reviews, as well as those restricted to reporting the prevalence of a single genotype or animal-adapted members of Mtb and that were not published in English or Persian. In studies with overlapping data, times, and sample collection locations, we prioritized the study that provided the most detailed genotyping information. We did not impose restrictions based on publication date.

It should be noted that the WHO revised the definitions of XDR-TB in 2021. According to the updated definition, XDR-TB refers to a MDR-TB that also shows resistance to a fluoroquinolone and at least one Group A agent (bedaquiline or linezolid). As all pre-/XDR studies included in our meta-analysis predate 2021, XDR-TB in this review was defined according to the pre-2021 WHO definition (an MDR strain with additional resistance to a fluoroquinolone and at least one of the injectable agents, amikacin, kanamycin, or capreomycin). We retained the earlier WHO definitions reported by each study and interpreted the pooled results, acknowledging this shift.

## Data extraction and quality assessment

The following data were extracted from the eligible studies:

First author, publication year, study location, typing method, detected Mtb genotypes, prevalence of MDR and pre-/XDR genotypes, sample sizes, and population characteristics (pulmonary/extrapulmonary, nationality) (Table 1). Two independent reviewers extracted the data.

**Table 1:** Characteristics of included studies in this meta-analysis

First author	Location	Year of publication	Typing method	Genotypes	Total genotypes	Total MDR	Total sample	Genotypes	Total genotypes	Total pre-XDR/XDR	Total sample	Type of sample	Nationality
Bakhtiyariniya [17]	Khuzestan	2022	24-MIRUVNTR	CAS,NEW1,LAM, EAI	CAS:4/NEW1:1/LAM:1/EAI:1	10	29	-	-	-	-	PTB	Iranian
Ebrahimzadeh [18]	Tehran	2021	24-MIRUVNTR	Beijing,Cameroon	Beijing:2/Cameroon:1	3	50	-	-	-	-	PTB	Iranian
Ramazanzadeh [19]	Ardabil, Hamadan, Qazvin, Tabriz and Kurdistan	2020	Spoligo	NEW1,U	NEW1:4,U:4	12	47	-	-	-	-	PTB	Iranian
Kardan-Yamchi [20]	IRAN	2020	WGS	Beijing, CAS, URAL, LAM, NEW1	Beijing:5/CAS:6/URAL:2/LAM:3/NEW1:1	17	35	Beijing, LAM, NEW1	Beijing:7/LAM:2/NEW1:4	13	35	PTB/EPTB	Iranian/immigrants
Kousha [21]	East Azerbaijan, Kurdistan, and Kermanshah	2020	IS6110 RFLP	Beijing	Beijing:2	2	64	-	-	-	-	PTB	Iranian
Mansoori [22]	Golestan	2020	Spoligo	Beijing	Beijing:2	2	166	-	-	-	-	PTB	Iranian
Vaziri [23]	Tehran	2019	WGS	Beijing	Beijing:3	3	38	Beijing, CAS, NEW1	Beijing:11/CAS:1/NEW1:1	13	38	PTB	Iranian
Hadifar [24]	Tehran	2019	24- loci MIRU-VNTR/Spoligo	Beijing, CAS, NEW1	Beijing:2/CAS:1/NEW1:2	5	172	Beijing, CAS, T	Beijing:3/CAS:1/T:1	5	172	PTB/EPTB	Iranian
Riyahi Zaniani [25]	Isfahan	2018	24-MIRUVNTR	Beijing, LAM, CAS	Beijing :5/LAM:2/CAS:1	8	18	-	-	-		PTB/EPTB	Iranian
Khosravi [26]	Ahvaz, Shiraz, Gorgan, Kermanshah, Mashhad and Qom	2017	12-MIRUVNTR	Beijing,NEW1,CAS, LAM	Beijing :7/NEW1 :1/CAS:1/LAM:2	22	100	-	-	-		PTB	Iranian
Khosravi [27]	Ahvaz, Shiraz, Gorgan, Kermanshah, Mashhad, Kashan, Tehran and Qom	2017	12-MIRUVNTR	Beijing,Haarlem,Uganda, Bovis	Beijing :9/Haarlem:3/Uganda :1/Bovis:1	22	88	-	-	-		PTB	Iranian
Ravansalar [28]	Khorasan	2017	12-MIRUVNTR/Spoligo	Beijing	Beijing:3	3	140	-	-	-	31	PTB	Iranian/immigrants
Manson [29]	Global	2017	WGS	Beijing, CAS,T1	Beijing:1/CAS:1/T1:3	5	31	Beijing,CAS,T1,Haarlem	Beijing:19/CAS:1/T1:4/Haarlem:2	26		-	Iranian
Rezaei [30]	Tehran, Zahedan, Isfahan and Khorasan	2016	Spoligo	Beijing, CAS, NEW1,T1, H37Rv	Beijing:3/CAS:1/NEW1:1/T1:1/,H37Rv:3	9	23	-	-	-		PTB	Iranian
Khanipour [31]	Tehran	2016	24-MIRUVNTR/Spoligo	Beijing, CAS, T1, Haarlem	Beijing:7/CAS:1/T1:1/Haarlem:6	15	23	Beijing, Haarlem,T1	Beijing:2/Haarlem:5/T1:1	8	23	PTB	Iranian
Rezaei [32]	Tehran, Zahedan, Isfahan and Khorasan	2016	Spoligo	Beijing, CAS, NEW1,URAL	Beijing:3/CAS:1/NEW1:3/URAL:3	10	20	-	-	-		PTB	Iranian
Zamani [33]	Hormozgan	2016	15-MIRU-VNTR/Spoligo	T1, MANU2	T1:2/MANU2:1	3	38	-	-	-		PTB	Iranian
Kazemian [34]	Tehran, Mashhad, Kermanshah and Zahedan	2015	Spoligo	Beijing, CAS, NEW1, LAM, T, EAI, MANU2,H37Rv	Beijing:13/CAS:4/NEW1:9/LAM:3/T:2/EAI:1/MANU2:2/H37Rv:3	39	40	Beijing	Beijing:1	1	40	PTB	Iranian
Kardan-Yamchi [35]	IRAN	2015	Spoligo	Beijing,CAS, NEW1, LAM, EAI,H37Rv	Beijing: 7/CAS:2/NEW1:1/LAM:3/EAI:1/H37Rv:4	20	31	Beijing	Beijing:1	1	31	PTB	Iranian
Sharifpour [36]	Tehran	2014	Spoligo	CAS,Beijing, Haarlem,T1	Beijing:4/CAS:3/Haarlem:3/T1:3	30	190	-	-	-		PTB	Iranian
Varahram [37]	Tehran	2014	Spoligo	Beijing,CAS,Haarlem, LAM,T, EAI, MANU,U	Beijing: 10/CAS:1/Haarlem:2/LAM:1/T:1/EAI:5/MANU:2/U:1	23	151	-	-	-		PTB	Iranian/immigrants
Haeili [38]	Tehran, Alborz, Sistan-Baluchestan, Hormozgan, and Kermanshah	2013	Spoligo	Beijing, CAS, URAL	Beijing: 3/CAS:1/URAL:5	15	291	-	-	-		PTB	Iranian
Mozafari [39]	Tehran	2012	MIRUVNTR/Spoligo	Beijing	Beijing:7	36	105	-	-	-		PTB	Iranian
Zaker Bostanabad [40]	Tehran	2011	Spoligo	Beijing, CAS, EAI, U	Beijing:1/CAS:2/EAI:3/U:1	7	149	-	-	-		PTB	Iranian
Jafarian [41]	Tehran	2010	12-MIRUVNTR/Spoligo	Beijing,CAS,Haarlem,Uganda,Cameron,Ghana,Bovis	Beijing:1/CAS:5/Haarlem:6//LAM:4/Uganda:2/Cameron:2/Ghana:1/Bovis:1	30	60	-	-	-		PTB	Iranian/immigrants
Velayati [42]	Tehran	2009	Spoligo	-	-	-	-	Beijing,CAS,Haarlem,EAI	Beijing:5/CAS:4/Haarlem:9/EAI:5	23	23	PTB	Iranian/immigrants
Doustdar [43]	Tehran	2009	IS6110 RFLP/Spoligotyping	Beijing, CAS, EAI, Haarlem	Beijing:6/CAS:2/EAI:1/Haarlem:5	14	34	-	-	-		PTB	Iranian
Ahmadi [44]	Tehran	2009	Spoligo	Beijing, CAS, EAI, Haarlem,T1,U	Beijing:6/CAS:7/EAI:8/Haarle:3/T1:2/U:2	28	238	-	-	-		PTB	Iranian/immigrants
Doustdar [45]	Tehran	2008	Spoligotyping	Beijing, CAS, Haarlem	Beijing: 3/CAS:1/Haarlem:1	5	30	-	-	-		PTB	Iranian
Masjedi [46]	Tehran	2008	Spoligo	Beijing, Haarlem	Beijing:4/Haarlem:8	12	65	-	-	-		PTB/EPTB	Iranian/immigrants
Masjedi [47]	Tehran	2007	IS6110 RFLP/Spoligotyping	CAS, Haarlem, EAI	CAS:2/Haarlem:13/EAI:14	31	31	-	-	-		PTB	Iranian
Farnia [48]	Tehran	2006	Spoligo	Beijing, CAS,Haarlem, EAI,X,T1	Beijing:52/ CAS:32/Haarlem:85/EAI:21/X:12/ T1:11	263	263	-	-	-		PTB/EPTB	Iranian/immigrants
Amirmozafari [49]	Tehran	2006	Spoligo	Beijing	Beijing:14	76	439	-	-	-		PTB/EPTB	Iranian/immigrants

PTB: Pulmonary Tuberculosis, EPTB: Extrapulmonary Tuberculosis, WGS: Whole-Genome Sequencing, MDR: Multidrug resistant, XDR: Extensively drug-resistant.

Two authors using a modified Newcastle–Ottawa Scale (NOS) independently evaluated the quality of each included study [50]. Evaluation was based on participant selection, design and analysis comparability, and outcome ascertainment. The maximum possible score on this method is nine (categorized as high quality). Low-quality studies were excluded from further analysis.

## Statistical analysis

The point estimate and 95% confidence interval (CI) were calculated to determine the prevalence of Mtb genotypes among Iranian MDR/XDR-TB patients. Heterogeneity across the studies was assessed using the I^2^ statistic, with a threshold of ≥ 50%, and the Chi-square test, with a P-value < 0.10, was applied to indicate significant heterogeneity [51]. Random-effects meta-regression was conducted to explore the sources of heterogeneity. The Egger regression asymmetry test was employed to assess potential publication bias, with a significance threshold of 5% [52]. A sensitivity analysis was carried out to evaluate the potential influence of the effect of small sample size study (*n* < 5 and *n* < 10) on the overall findings. Finally, the subgroup analyses were conducted by year of publication. All statistical analyses and graphical representations were conducted using the meta package in R (v 4.1.1). A two-sided P-value of ≤ 0.05 was considered statistically significant unless otherwise specified.

## Results

### Search results and studies’ characteristics

**Fig. 1:**
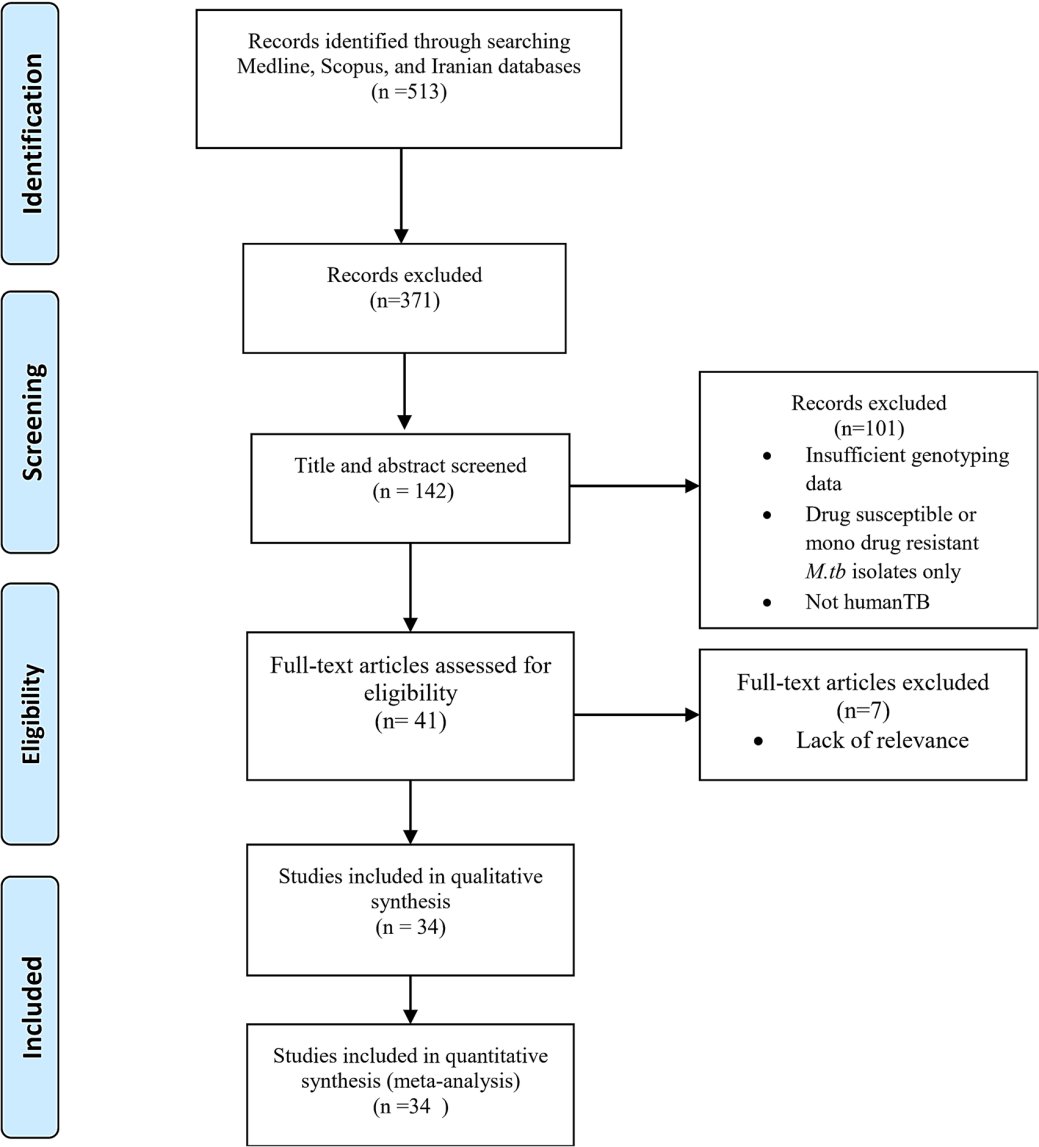
Flow diagram of meta-study

A total of 513 papers were screened, and based on title and abstract, 371 were excluded for being irrelevant or duplicates (Fig. 1). Forty-one studies were eligible for full-text review, of which seven were subsequently excluded based on the criteria. Ultimately, 34 studies were selected to assess the prevalence of Mtb genotypes among Iranian MDR/XDR-TB cases. Of these, eight studies focused on pre-/XDR isolates, while the remaining studies provided data on MDR-TB. The publication year of the reviewed studies among MDR genotypes ranged from 2006 to 2022, whereas the studies on pre-/XDR-TB spanned 2009–2020 (before the 2021 WHO definition update).

All papers included in the systematic review assessing the prevalence of MDR genotypes were cross-sectional studies, with sample sizes ranging from two to 263. For studies investigating the prevalence of pre-/XDR genotypes, the highest sample size was 26. Of the 34 conducted studies, five included both Iranian participants and several immigrants. In both MDR and pre-/XDR groups, spoligotyping and MIRU-VNTR typing were mainly used for genotyping (Table 1). The detailed information regarding the included studies is presented in Table 1.

### Prevalence rate of MDR/XDR-Mtb genotypes

Based on the results of the 34 included studies, there were 780 MDR-Mtb genotypes and 90 pre-/XDR-Mtb genotypes. Random or fixed effects meta-analysis results indicated Haarlem as 28.5% (95% CI: 24.6–32.8), Beijing as 25.6% (95% CI: 22.5–28.9), and URAL as 23.8% (95% CI: 13.3–38.9) as the predominant MDR genotypes. MANU, T, and Uganda were identified as Mtb genotypes with the lowest prevalence ( < 8%). More details about the overall prevalence are presented in Table 2.

**Table 2:** The pooled prevalence of MDR/XDR MTBC genotypes in TB patients

Type	Lineage	Genotype	Study numbers	Pooled prevalence of genotype			Heterogeneity				Publication bias	
				Prevalence%	(Number)	(95%CI)%	**I**^**2**^	Q		*p***-value**	Begg’s *p***-value**	Egger’s *p*-value
MDR	L4	Haarlem	11	28.5%	135	(24.6–32.8)%	**63.7%**	27.56		**0.002**	0.242	0.266
	L2	Beijing	28	25.6%	185	(22.5–28.9) %	33.6%	40.63		0.0447	0.065	0.002
	L4	URAL	3	23.80%	10	(13.3–38.9)%	7.10%	2.15		0.341	0.117	0.402
	L4	NEW1	9	15.97%	23	(10.8–22.9)%	27.5%	11.3		0.2	0.404	0.091
	L4	H37Rv	3	14.70%	10	(8.1–25.2)%	49.50%	3.9		0.138	0.602	0.669
		U	3	14.9%	7	(7.3–28.1)%	48.9%	3.91		0.141	0.602	0.691
	L3	CAS	21	13.04%	79	(10.6–15.9)%	11.4%	22.58		0.309	0.715	0.898
	L1	EAI	9	12.64%	55	(8.6–26.2)%	**80.8%**	41.73		** < 0.001**	0.531	0.904
	L4	LAM	8	11.24%	19	(7.3–16.9)%	0%	3.95		0.78	0.999	0.532
	L4	Cameroon	2	9.10%	3	(3.0–24.7)%	46.20%	1.86		0.173	-	-
	L1	MANU	3	7.70%	5	(3.2–17.2)%	18.10%	2.4		0.295	0.117	0.204
	L4	T	9	6.26%	26	(4.3–9.0)%	**63.80%**	22.1		**0.005**	0.211	0.065
	L4	Uganda	2	5.80%	3	(1.9–16.4)%	0%	0.1		0.747	-	-
		Bovis	2	3.85%	2	(1.0–14.1)%	0%	0.1		0.823	-	-
Pre-/XDR	L2	Beijing	8	57.60%	49	(35.8–76.8)%	**61.50%**	18.2		**0.011**	0.706	0.728
	L4	Haarlem	3	30.20%	16	(9.8–63.3)%	**77.70%**	9		**0.011**	0.602	0.822
	L4	NEW1	2	19.20%	26	(8.2–38.7)%	48.50%	1.9		0.164	-	-
	L4	T	3	15.40%	39	(7.1–30.3)%	0%	0.1		0.936	0.602	0.917
	L3	CAS	4	10.5%	67	(5.1–20.3)%	0%	2.6		0.462	0.999	0.430

**Fig. 2:**
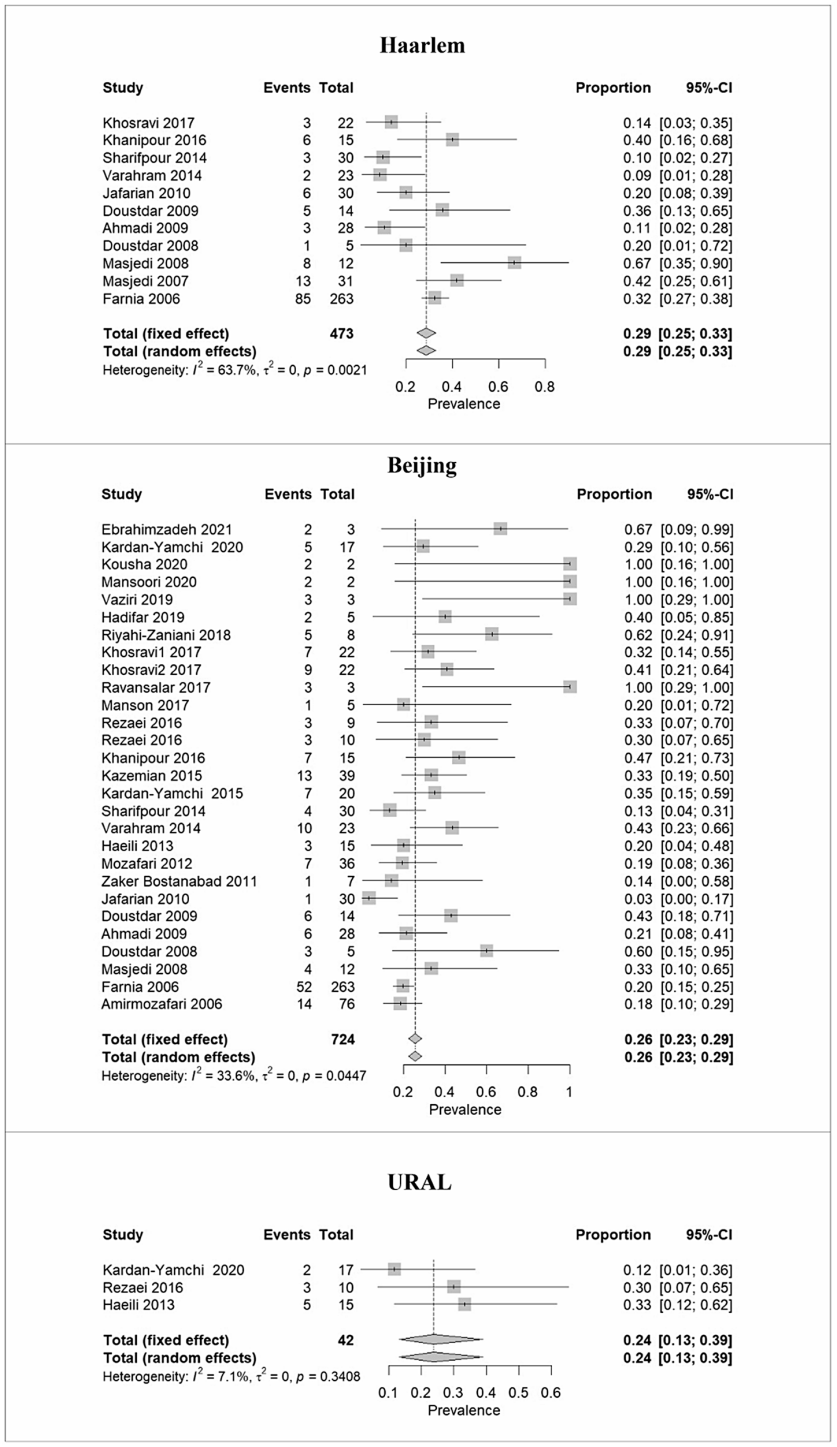
Forest plots illustrate the prevalence of predominant Mtb genotypes among MDR-TB cases

**Fig. 3:**
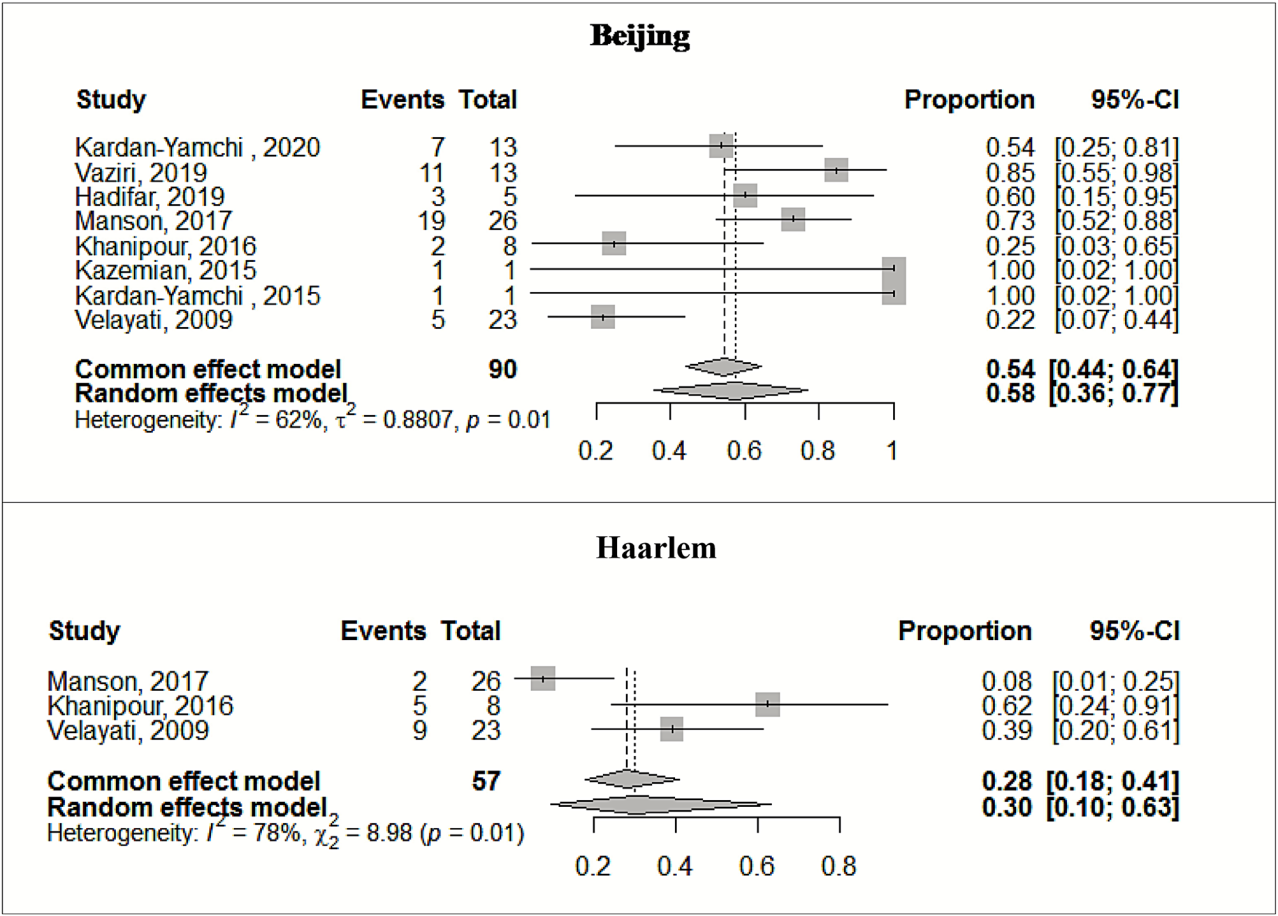
Forest plots present the prevalence of predominant Mtb genotypes among pre-/XDR-TB cases

Beijing genotype was particularly dominant in pre-/XDR cases (57.6%, 95% CI: 35.8–76.8), followed by Haarlem (30.2%, 95% CI: 9.8–63.3) and NEW1 (19.2%, 95% CI: 8.2–38.7) (Table 2). Notably, CAS was revealed as the genotype with the lowest prevalence in Iranian pre-/XDR-TB cases (10.5%, 95% CI: 5.1–20.3). The forest plots in Figs. 2 and 3 illustrate the prevalence of dominant genotypes in both studied groups, providing a visual representation of the data.

### Subgroup analysis

The result of year grouping, significant changes were observed for the Beijing (pre-/XDR-TB; *p* = 0.006) and EAI (MDR-TB; *p* = 0.027) and genotype. The EAI genotype showed a significant decrease after 2015, whereas the prevalence of Beijing lineage with higher resistance levels (Pre-/XDR) significantly increased during the same period (Table 3). The highest and lowest prevalence of MDR genotypes in the studies that were published before 2015 were related to Haarlem (24.3%, 95% CI: 14.7–37.5) and T (4.94%, 95% CI: 3.1–7.8). In contrast, after 2015, Beijing (40%, 95% CI: 33.1–47.2) and EAI (4.35%; 95% CI: 3.1–7.8) were observed with the highest and lowest prevalence genotypes. Similarly, in pre-/XDR-TB cases, Beijing showed growing prevalence over time (Supplementary file).

**Table 3:** Subgroup analysis of *M. tb* genotypes by study year

Type	Variables		Estimate (95%CI)	P-value
	Beijing	Before 2015	Ref	< 0.001
MDR		After 2015	0.94 (0.58, 1.30)	
	CAS	Before 2015	Ref	0.558
		After 2015	0.15 (−0.36, 0.68)	
	Haarlem	Before 2015	Ref	0.978
		After 2015	−0.19 (−1.42, 1.38)	
	EAI	Before 2015	Ref	0.027
		After 2015	−1.84 (−3.47, −0.21)	
	T	Before 2015	Ref	0.082
		After 2015	1.15 (−0.14, 2.45)	
Pre-/XDR	Beijing	Before 2015	Ref	0.006
		After 2015	1.93 (0.55, 3.31)	

### Publication bias and sensitivity analysis

Several cases in Table 2 showed significant heterogeneity in prevalence across the included studies, as indicated by Q test results (Cochran’s Q *p* < 0.05; I^2^ > 50%). However, the result of Egger’s linear regression and Begg’s tests showed that publication bias was not statistically significant in this meta-analysis, except for MDR–Beijing (Egger *p* = 0.002) and a borderline signal for MDR–T (*p* ≈ 0.065). (Table 2). We conducted a sensitivity analysis with two scenarios: excluding studies with sample sizes less than 5 and then excluding studies with sample sizes less than 10. The results showed no significant difference or substantial impact on the pooled prevalence estimates. These findings are detailed in the supplementary file.

## Discussion

*M. tuberculosis* drug resistance is a major public health issue, jeopardizing WHO‘s End TB goals [53]. Understanding the transmission dynamics of drug-resistant Mtb genotypes and tailoring effective regional control strategies are feasible through targeted molecular surveillance. Accordingly, the current study aimed to provide a comprehensive overview of the diversity and pooled prevalence of MDR and pre-/XDR Mtb genotypes in Iran through a systematic review and meta-analysis.

Our analysis revealed that Haarlem (28.5%, 95% CI: 24.6–32.8), Beijing (25.6%, 95% CI: 22.5–28.9), and URAL (23.8%, 95% CI: 13.3–38.9) were the predominant genotypes in the MDR-TB population, respectively, and among the pre-/XDR-TB cases, the Beijing genotype had the highest frequency (57.6%, 95% CI: 35.8–76.8). The results are broadly consistent with global and regional trends and can contribute to informing local health planning [54, 55].

Studies have shown that Beijing strains are associated with low cure rates, treatment failures, and faster transmission, in addition to their greater capability to develop MDR and XDR. The association of Beijing with drug resistance has been linked to compensatory mutations that mitigate fitness costs, facilitating persistent transmission [56]. Reports from China, India, Russia, and the Middle Eastern countries have repeatedly emphasized that the Beijing genotype is responsible for a significant proportion of MDR/XDR-TB cases [13, 55, 57–60]. In this context, the prominence of Beijing among Iranian pre-/XDR- and MDR-TB populations may signify its historical introduction through migration, particularly cross-border migration from high-burden neighbours (notably Afghanistan and Pakistan), successful local adaptation and clonal expansion, leveraged by its enhanced transmissibility and propensity for accumulating drug resistance mechanisms. However, the hypotheses are plausible; genomic studies are needed to confirm these associations. Further, year grouping identified a significant heterogeneity in Beijing genotype (*p* < 0.001), whereas the prevalence of this genotype was increasing after 2015 (MDR-TB: 40.0%, pre-/XDR-TB: 65.67%). Based on the increasing prevalence trend of this genotype and its pathogenic capability, local tracking dynamics of drug-resistant Mtb population through molecular characterisation and genomic typing are critical to improving our ability to manage local MDR/XDR-TB.

Haarlem genotype is also a major component of the global MDR-TB landscape, notable for its prevalence in diverse regions [61, 62]. Reports from Iran and neighbouring countries (e.g., Turkey, Pakistan) document this genotype among drug-resistant patients. Additionally, an increase in prevalence of Haarlem from 2% to 70% documented for Afghan immigrants in Iran [14, 63]. The prominence of Haarlem in both MDR and pre-/XDR-TB in our meta-analysis aligns with a previous report [63]from Iran showing the dominating Haarlem genotype in the MDR-TB population. Persistence in the prevalence of Haarlem could potentially reflect its adaptation, transmission and survival capacity, and potential biological advantages under selective drug pressure.

URAL and NEW1 genotypes, which were linked to MDR- and pre-XDR-TB populations with high pooled proportion in our analysis, are not globally distributed. Still, the association of these genotypes with MDR and an increase in their prevalence has garnered considerable attention [14, 64]. Intriguingly, the phylogeography of NEW1 is specific to Iran; however, the pattern of distribution changes remarkably [14]. The high frequency of these genotypes in MDR and XDR cases underscores an emerging health concern in our region.

In the pre-/XDR-TB population, the CAS genotype was less prevalent (10.5%, 95% CI: 5.1–20.3%). From 2004 onward, the proportion of CAS represents a noticeable change in MDR/XDR [65]. Given the predominant presence of the CAS genotype around the Indian Ocean and West Asia, the importation of this strain by people from these regions, combined with its biological capabilities for local adaptation, may justify the presence of this genotype. Other genotypes, such as LAM, T, Cameroon, and EAI, with different proportions, were also observed in our meta-analysis. This high genetic diversity and the variation in the prevalence of MDR/XDR genotypes reflect a combination of numerous factors, including migration patterns, treatment behaviours, methodological differences (e.g., MIRU-VNTR), the biological capabilities of genotypes, and disease control policies. The transition toward all-oral regimens and wider use of bedaquiline and linezolid may influence selection pressures and transmission dynamics; however, causal inferences are beyond the scope of this review.

This study has some limitations; first, small sample sizes in some studies and relatively wide confidence intervals, particularly those focusing on pre-/XDR-TB cases, may have affected the precision of the pooled prevalence estimates and limited generalizability. Second, all pre-/XDR-TB data in this review predate the 2021 WHO revision of definitions; therefore, our estimates reflect prior criteria and may not be fully comparable with data generated under the updated definitions. Third, substantial heterogeneity likely arose from moderators such as geography, age, and nationality, which were not feasible due to incomplete reporting across studies. Finally, as some DR-TB studies reported drug susceptibility patterns without corresponding genotyping, the pooled proportions may not capture the full underlying genotype distribution in Iran.

## Conclusion

In conclusion, the results of this study highlighted the significant genotypic diversity within the MDR- and pre-/XDR-TB populations, particularly the high prevalence of Haarlem and Beijing genotypes. The situation of Iran at the crossroads of high TB burden regions, as well as its migrant and refugee population, has created complex conditions for the spread of drug-resistant genotypes. This emphasizes the urgent need for continuous molecular monitoring programs and genotype-informed treatment and control strategies in the country. To enhance treatment effectiveness and better manage the MDR-/XDR-TB epidemic in Iran, it is crucial to improve molecular diagnostic capabilities and integrate methods like 24-locus MIRU-VNTR typing into routine surveillance, strengthening detection and systematic contact tracing, with prioritization of high-risk genotypes to inform targeted interventions.

## Electronic supplementary material

Below is the link to the electronic supplementary material.

Supplementary Material 1

## Data Availability

No datasets were generated or analysed during the current study.

## Abbreviations

TB: Tuberculosis
Mtb: *Mycobacterium tuberculosis*
MDR: Multidrug-resistant
pre-XDR: Pre-extensively drug-resistant
XDR: Extensively drug-resistant
DR-TB: Drug-resistant tuberculosis
MIRU-VNTR: Mycobacterial interspersed repetitive unit-variable number tandem repeat
IS6110-RFLP: Insertion sequence 6110-restriction fragment length polymorphism
WGS: Whole genome sequencing
NOS: Newcastle–ottawa scale
