# Supplementary material for: Genotypic diversity of multi- and extensively drug-resistant Mycobacterium tuberculosis in Iran: a systematic review and meta-analysis
Source: BMC Infect Dis. 2025 Dec 15;26:100. doi: 10.1186/s12879-025-12299-y (PMC12822001; doi:10.1186/s12879-025-12299-y)
Supplement: Supplementary file 1 — Supplementary Material 1 [file 12879_2025_12299_MOESM1_ESM.docx]

Meta-analysis results by genotype for the studies published before and after 2015

| **Type** | **Lineage** | **Genotype** | **Study numbers** | **Pooled prevalence of genotype** | | |  | **Heterogeneity** | | |  | **Publication bias** | |
| --- | --- | --- | --- | --- | --- | --- | --- | --- | --- | --- | --- | --- | --- |
|  |  |  |  | **Prevalence%** |  | **(95%CI)%** |  | **I^2^** | **Q** | ***p*-value** |  | **Begg’s**  ***p*-value** | **Egger’s *p*-value** |
| Before 2015 | | | | | | | | | | | | | |
| **MDR** | L4 | Haarlem | 9 | 24.3% |  | (14.7-37.5)% |  | **67.0%** | 24.23 | **0.002** |  | 0.531 | 0.335 |
|  | L2 | Beijing | 12 | 20.59% |  | (17.4-24.2) % |  | 46.2% | 20.45 | 0.039 |  | 0.999 | 0.582 |
|  | L3 | CAS | 10 | 12.56% |  | (9.8-15.9)% |  | 0.0% | 8.70 | 0.465 |  | 0.654 | 0.970 |
|  | L1 | EAI | 6 | 21.2% |  | (11.1-36.6)% |  | **86.0%** | 35.81 | **<0.001** |  | 0.851 | 0.369 |
|  | L4 | T | 4 | 4.94% |  | (3.1-7.8)% |  | **0.0%** | 2.14 | **0.543** |  | 0.999 | 0.429 |
| **Pre-/XDR** | L2 | Beijing | 1 | 21.74% |  | (9.3-42.8)% |  | - | - | - | - | - | - |
| After 2015 | | | | | | | | | | | | | |
| **MDR** | L4 | Haarlem | 2 | 24.4% |  | (10.6-46.8)% |  | **68.0%** | 3.13 | **0.077** |  | - | - |
|  | L2 | Beijing | 16 | 40.0% |  | (33.1-47.2) % |  | 0.0% | 5.56 | 0.986 |  | 0.015 | 0.003 |
|  | L3 | CAS | 11 | 14.38% |  | (9.7-20.7)% |  | 21.6% | 12.76 | 0.237 |  | 0.347 | 0.152 |
|  | L1 | EAI | 3 | 4.35% |  | (1.4-12.6)% |  | **0.0%** | 0.33 | **0.615** |  | 0.117 | 0.122 |
|  | L4 | T | 5 | 17.56% |  | (4.8-47.1)% |  | **68.8%** | 12.21 | **0.012** |  | 0.327 | 0.361 |
| **Pre-/XDR** | L2 | Beijing | 7 | 65.67% |  | (53.6-76.0)% |  | **23.9%** | 7.89 | **0.246** |  | 0.999 | 0.895 |

Sensitivity analysis results after excluding studies with sample size of less than 5 (≥5)

| **Type** | **Lineage** | **Genotype** | **Study numbers** | **Pooled prevalence of genotype** | | |  | **Heterogeneity** | | |  | **Publication bias** | |
| --- | --- | --- | --- | --- | --- | --- | --- | --- | --- | --- | --- | --- | --- |
|  |  |  |  | **Prevalence%** | **(Number)** | **(95%CI)%** |  | **I^2^** | **Q** | ***p*-value** |  | **Begg’s**  ***p*-value** | **Egger’s *p*-value** |
| **MDR** | L4 | Haarlem | 11 | 28.5% | 135 | (24.6-32.8)% |  | **63.7%** | 27.56 | **0.002** |  | 0.242 | 0.266 |
|  | L2 | Beijing | 23 | 24.3% | 173 | (21.3-27.6) % |  | 43.0% | 38.60 | 0.0157 |  | 1.000 | 0.092 |
|  | L4 | URAL | 3 | 23.80% | 10 | (13.3-38.9)% |  | 7.10% | 2.15 | 0.341 |  | 0.117 | 0.402 |
|  | L4 | NEW1 | 9 | 15.97% | 23 | (10.8-22.9)% |  | 27.5% | 11.03 | 0.200 |  | 0.404 | 0.091 |
|  | L4 | H37Rv | 3 | 14.70% | 10 | (8.1-25.2)% |  | 49.50% | 3.9 | 0.138 |  | 0.602 | 0.669 |
|  |  | U | 3 | 14.9% | 7 | (7.3-28.1)% |  | 48.9% | 3.91 | 0.141 |  | 0.602 | 0.691 |
|  | L3 | CAS | 21 | 13.04% | 79 | (10.6-15.9)% |  | 11.4% | 22.58 | 0.309 |  | 0.715 | 0.898 |
|  | L1 | EAI | 9 | 12.64% | 55 | (8.6-26.2)% |  | **80.8%** | 41.73 | **<0.001** |  | 0.531 | 0.904 |
|  | L4 | LAM | 8 | 11.24% | 19 | (7.3-16.9)% |  | 0% | 3.95 | 0.785 |  | 0.999 | 0.532 |
|  | L4 | Cameroon | 2 | 9.10% | 3 | (3.0-24.7)% |  | 46.20% | 1.86 | 0.173 |  | - | - |
|  | L1 | MANU | 2 | 6.45% | 4 | (2.4-15.9)% |  | 0.0% | 0.30 | 0.584 |  | - | - |
|  | L4 | T | 8 | 6.96% | 24 | (3.6-13.1)% |  | **52.80%** | 14.82 | **0.038** |  | 0.458 | 0.161 |
|  | L4 | Uganda | 2 | 5.80% | 3 | (1.9-16.4)% |  | 0% | 0.1 | 0.747 |  | - | - |
|  |  | Bovis | 2 | 3.85% | 2 | (1.0-14.1)% |  | 0% | 0.1 | 0.823 |  | - | - |
| **Pre-/XDR** | L2 | Beijing | 6 | 53.69% | 47 | (31.9-74.1)% |  | **72.50%** | 18.20 | **0.003** |  | 0.851 | 0.985 |
|  | L4 | Haarlem | 3 | 30.20% | 16 | (9.8-63.3)% |  | **77.70%** | 9.0 | **0.011** |  | 0.602 | 0.822 |
|  | L4 | NEW1 | 2 | 19.20% | 26 | (8.2-38.7)% |  | 48.50% | 1.9 | 0.164 |  | - | - |
|  | L4 | T | 3 | 15.40% | 39 | (7.1-30.3)% |  | 0% | 0.1 | 0.936 |  | 0.602 | 0.917 |
|  | L3 | CAS | 4 | 10.5% | 67 | (5.1-20.3)% |  | 0% | 2.6 | 0.462 |  | 0.999 | 0.430 |

Sensitivity analysis results after excluding studies with sample size of less than 10 (≥10)

| **Type** | **Lineage** | **Genotype** | **Study numbers** | **Pooled prevalence of genotype** | | |  | **Heterogeneity** | | |  | **Publication bias** | |
| --- | --- | --- | --- | --- | --- | --- | --- | --- | --- | --- | --- | --- | --- |
|  |  |  |  | **Prevalence%** | **(Number)** | **(95%CI)%** |  | **I^2^** | **Q** | ***p*-value** |  | **Begg’s**  ***p*-value** | **Egger’s *p*-value** |
| **MDR** | L4 | Haarlem | 10 | 28.6% | 134 | (24.6-32.8)% |  | **67.0%** | 27.31 | **0.001** |  | 0.179 | 0.307 |
|  | L2 | Beijing | 17 | 23.5% | 158 | (20.5-26.9) % |  | 46.4% | 29.80 | 0.0188 |  | 0.621 | 0.233 |
|  | L4 | URAL | 3 | 23.80% | 10 | (13.3-38.9)% |  | 7.10% | 2.15 | 0.341 |  | 0.117 | 0.402 |
|  | L4 | NEW1 | 7 | 15.38% | 20 | (10.2-22.6)% |  | 36.2% | 9.41 | 0.152 |  | 0.452 | 0.057 |
|  | L4 | H37Rv | 2 | 11.86% | 7 | (5.7-22.8)% |  | 44.20% | 1.79 | 0.180 |  | - | - |
|  |  | U | 2 | 15.7% | 6 | (4.7-41.1)% |  | 73.9% | 3.84 | 0.050 |  | - | - |
|  | L3 | CAS | 15 | 12.71% | 72 | (10.2-15.7)% |  | 33.1% | 20.93 | 0.103 |  | 0.234 | 0.620 |
|  | L1 | EAI | 8 | 12.15% | 52 | (9.4-15.6)% |  | **81.70%** | 38.33 | **<0.001** |  | 0.804 | 0.909 |
|  | L4 | LAM | 7 | 10.56% | 17 | (6.7-16.3)% |  | 0% | 2.70 | 0.845 |  | 0.652 | 0.219 |
|  | L4 | Cameroon | 2 | 9.10% | 3 | (3.0-24.7)% |  | 46.20% | 1.86 | 0.173 |  | - | - |
|  | L1 | MANU | 2 | 6.45% | 4 | (2.4-15.9)% |  | 0.0% | 0.30 | 0.584 |  | - | - |
|  | L4 | T | 6 | 5.03% | 26 | (3.3-7.7)% |  | **0.0%** | 2.21 | **0.820** |  | 0.851 | 0.277 |
|  | L4 | Uganda | 2 | 5.80% | 3 | (1.9-16.4)% |  | 0% | 0.1 | 0.747 |  | - | - |
|  |  | Bovis | 2 | 3.85% | 2 | (1.0-14.1)% |  | 0% | 0.1 | 0.823 |  | - | - |
| **Pre-/XDR** | L2 | Beijing | 4 | 58.94% | 42 | (31.8-81.5)% |  | **80.80%** | 15.65 | **0.001** |  | 0.999 | 0.737 |
|  | L4 | Haarlem | 2 | 19.83% | 11 | (5.4-51.5)% |  | **82.70%** | 5.8 | **0.016** |  | - | - |
|  | L4 | NEW1 | 2 | 19.20% | 26 | (8.2-38.7)% |  | 48.50% | 1.9 | 0.164 |  | - | - |
|  | L3 | CAS | 3 | 9.68% | 62 | (4.4-19.9)% |  | 12.5% | 2.29 | 0.318 |  | 0.602 | 0.233 |
